# Traitement endoscopique des complications biliaires de l'hydatidose hépatique

**DOI:** 10.11604/pamj.2015.22.67.7616

**Published:** 2015-09-25

**Authors:** Hanane Massit, Hassan Seddik, Hanane Basr, Ahmed Benkirane

**Affiliations:** 1 Service d'Hépato-Gastroentérologie II, Hôpital Militaire d'Instruction Med V, Rabat, Maroc

**Keywords:** Fistule biliaire, kyste hydatique du foie, sphinctérotomie endoscopique, biliary fistula, hydatid cyst of the liver, endoscopic sphincterotomy

## Abstract

L'objectif était d’évaluer l'efficacité du traitement endoscopique des complications biliaires de l'hydatidose hépatique. Ont été inclus 36 patients hospitalisés pour prise en charge endoscopique des complications biliaires du kyste hydatique du foie (KHF) sur une période de 13 ans, d'un âge moyen de 46 ans et avec une prédominance masculine dans 63% des cas. La prévalence des complications biliaires du KHF est de 2,3% dans notre série. La cholangiopancréatographie rétrograde endoscopique (CPRE) a permis d'objectiver une fistule kystobiliaire dans 35% des cas, des images lacunaires dans la voie biliaire principale (VBP) dans 70% des cas en rapport avec du matériel hydatique. La sphinctérotomie endoscopique (SE) a été réalisée chez tous les patients permettant l'extraction de matériel hydatique par ballonnet d'extraction ou par panier Dormia, ainsi qu'une dilatation de la VBP par bougie. L’évolution a été marquée par la disparition de l'ictère au bout de 5 à 12 jours en moyenne après le geste endoscopique et par le tarissement de la fistule biliaire externe au bout de 10 à 12 jours. Aucune complication liée au traitement endoscopique n'a été objectivée. Trois patients ont évolué vers une cholangite sclérosante qui s'est compliquée d'une cirrhose biliaire secondaire au bout de 3 ans. Le traitement endoscopique des complications biliaires d'hydatidose hépatique est une thérapie sûre et efficace avec une faible morbidité et mortalité.

## Introduction

Le kyste hydatique du foie (KHF) est une affection parasitaire due au développement de la forme larvaire du tænia du chien Echinococcus granulosus. Cette pathologie demeure fréquente et constitue un problème de santé publique dans les pays de forte endémie. Les pays du pourtour méditerranéen et plus particulièrement ceux du Maghreb sont considérés comme étant des pays d'endémie hydatique. Cette parasitose atteint essentiellement le foie (de 50 à 70% des cas); le poumon vient en seconde position (de 20 à 30% des cas) [1]. L'hydatidose hépatique peut causer une morbidité grave à cause de complications biliaires. La sphinctérotomie endoscopique (SE) et le drainage biliaire sont des méthodes très efficaces de prise en charge. Le but de notre étude est d’évaluer l'efficacité du traitement endoscopique des complications de l'hydatidose hépatique.

## Méthodes

Etude rétrospective menée au sein du service de gastro-entérologie II de l'Hôpital Militaire d'Instruction Mohammed V de Rabat sur une période de 13 ans: entre Janvier 2002 et Mars 2015. Ont été inclus 36 cas de KHF fistulisés dans les voies biliaires. La CPRE et la sphinctérotomie biliaire endoscopique ont été réalisée chez tous les patients. 16 fois en préopératoire et 20 fois en post opératoire. Le diagnostic de KHF fistulisé dans les voies biliaires a été porté devant les critères biologiques et morphologiques. Tous les patients ont bénéficié d'un examen physique complet, d'un bilan biologique et morphologique systématique. Une antibiothérapie prophylactique a été administrée par voie intraveineuse avant la procédure et poursuivie au moins cinq jours après. Toutes les CPRE ont été réalisées sous anesthésie générale par un endoscopiste expérimenté. Les endoscopes et les instruments utilisés étaient les suivants: Olympus JF-V260 et duodénoscopes Olympus FTCE-240; sphinctérotome triple lumière (Tri-tome^®^_ COOK^®^ TRI-25); fil-guide (Metro^®^ 35-480- COOK^®^); panier de Dormia (The Web^®^ Extraction Baskets-COOK^®^); ballonnet d'extraction (Tri-Ex^®^- COOK^®^); bougie 5-10F; drain naso-biliaire (ENBD-7- COOK^®^). Nous avons analysé les données épidémiologiques des malades, les résultats et le succès de la CPRE, ainsi que la morbidité et la mortalité post sphinctérotomie endoscopique.

## Résultats

**Figure 1:**
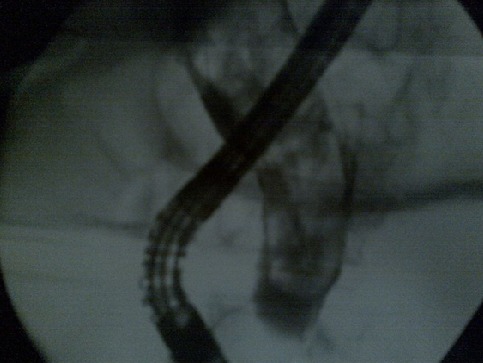
Image lacunaire dans la voie biliaire principale; matériel hydatique dans le cholédoque

**Figure 2:**
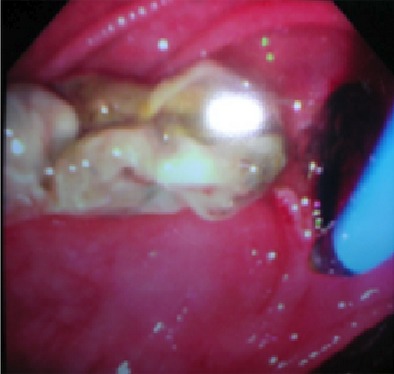
Extraction du matériel hydatique; image endoscopique

Sur un ensemble de 1550 CPRE réalisées pendant la période d’étude, l'indication était un KHF rompu dans les voies biliaires dans 36 cas, soit 2,3% des indications de la CPRE dans notre série. L’âge moyen des patients était de 46 ans, avec une prédominance masculine dans 63% des cas. La symptomatologie clinique était marquée par une fistule biliaire externe persistante en post opératoire dans 35% des cas, un ictère rétentionnel dans 25% des cas et un syndrome angiocholitique dans 40% des cas. Tous les patients ont présentés une cytolyse et/ou une cholestase biologique. Une échographie abdominale et une Tomendositométrie abdominale ont été réalisé chez tous les patients, elles ont objectivé des lésions kystiques chez tous les patients, une dilatation des voies biliaires intra et/ou extrahépatiques dans 77,7%, la présence du matériel hydatique dans la voie biliaire principale (VBP) chez 36% des patients. La CPRE a été réalisée chez tous les patients: 16 fois en préopératoire et 20 fois en post opératoire. Elle a permis d'objectiver une fistule kysto-biliaire dans 35% des cas, des images lacunaires dans la VBP (Figure 1) dans 70% des cas en rapport avec du matériel hydatique. Une sténose cholédocienne post opératoire a été rapportée chez un patient. La sphinctérotomie biliaire endoscopique a été réalisée chez tous les malades, avec extraction de matériel hydatique (Figure 2) par ballon dans 29 cas et par sonde de Dormia dans 7 cas. Une dilatation de la VBP à la bougie a été réalisée dans un seul cas. Alors qu'un drain naso-biliaire a été mis en place chez 7 patients permettant le drainage de la cavité résiduelle. L’évolution a été marquée par la disparition de l'ictère au bout de 5 à 12 jours en moyenne après le geste endoscopique et par le tarissement de la fistule biliaire externe au bout de 10 à 12 jours. Aucune complication liée au traitement endoscopique n'a été objectivée. Trois patients ont évolué vers une cholangite sclérosante qui s'est compliquée d'une cirrhose biliaire secondaire au bout de 3 ans.

## Discussion

Les kystes hydatiques du foie s'ouvrent dans les voies biliaires dans 2,5 à 6% des cas [2]. Dans notre série elle est de 2,3%. Dans sa forme typique, l'ouverture dans les voies biliaires se manifeste par une angiocholite aiguë dans 2/3 des cas [3] dans notre étude 40% des cas. La fréquente– 4257; stulisation du kyste hydatique dans les voies biliaires pose des problèmes de prise en charge: il est impossible d'injecter des produits scolicides et la chirurgie hépatique n'est pas dénuée de risques. Le traitement endoscopique dans cette situation peut être une alternative [4]. Le traitement chirurgical comporte un taux non négligeable de complications (38% des cas) à type de fuite biliaire, infection de la cavité résiduelle, récidive ou dissémination de la maladie, nécessitant souvent une réintervention. La mortalité s’élève à 4,5% dans la série de Zaouche et al. [5]. La SE a pour but d'assurer un meilleur drainage biliaire interne avec écoulement préférentiel de la bile vers le duodénum, favorisant ainsi la cicatrisation de la fistule biliaire. Plusieurs auteurs [6–10] ont rapporté l'intérêt de la SE associée ou non à d'autres procédés dans le traitement des complications post-opératoires du kyste hydatique du foie. L'utilisation du drainage naso-biliaire et l'insertion d'endoprothèse a été rapporté [11–13]. Les indications du traitement endoscopique sont classiquement les suivantes [4, 14]: à visée préopératoire en cas de vésicules filles ou de débris hydatiques dans l'arbre biliaire; sténoses bénignes postopératoires de l'ampoule de Vater; fistule biliaire externe post chirurgicale; débris intrabiliaires résiduels; cholangite sclérosante secondaire. Les données de plusieurs séries indiquent que le délai pour la fermeture des fistules externes est de 3 à 43 jours après SE [9, 10, 15]. Dans notre étude, la SE a permis, chez tous nos patients, l'inversion du flux biliaire avec drainage préférentiel vers le duodénum et tarissement de la fistule biliaire externe au bout de 10 à 12 jours, évitant ainsi le recours à une réintervention. Les résultats étaient satisfaisants chez tous les malades. Un complément de traitement par lavage des voies biliaires au sérum salé hypertonique a été réalisé, comme dans la série de Singh et al [16], malgré le risque potentiel de cholangite sclérosante secondaire, dans notre étude trois patients; soit 3,8% des cas; ont évolué vers une cholangite sclérosante qui s'est compliquée d'une cirrhose biliaire secondaire au bout de 3 ans.

## Conclusion

La sphinctérotomie endoscopique constitue un progrès thérapeutique dans les complications biliaires de l’échinococcose hépatique. C'est un traitement innovant qui a déjà montré son ef'cacité et son innocuité. Elle permet d’écourter le séjour post-opératoire et d’éviter une réintervention, souvent difficile et hémorragique.
